# Effects of immediate versus delayed frozen embryo transfer in high responder patients undergoing freeze-all cycles

**DOI:** 10.1186/s12884-021-03919-x

**Published:** 2021-06-28

**Authors:** Na Zuo, Yingzhuo Gao, Ningning Zhang, Da Li, Xiuxia Wang

**Affiliations:** 1 Center of Reproductive Medicine, Shengjing Hospital of China Medical University, Shenyang, 110004 China; 2 Department of Obstetrics and Gynecology, Shengjing Hospital of China Medical University, Shenyang, 110004 China

**Keywords:** High ovarian response, Frozen embryo transfer, Timing, GnRH analogue, In vitro fertilization, Pregnancy outcomes

## Abstract

**Background:**

Frozen embryo transfer (FET) can greatly improve the pregnancy outcomes for high responder patients. However, it is not known whether the timing of FET is a risk factor on pregnancy outcomes in high responder patients undergoing freeze-all cycles.

**Methods:**

A retrospective cohort study to compare the pregnancy outcomes of the immediate and delayed FET groups in high responder patients undergoing freeze-all cycles. The two groups were defined as that FET took place either within the first menstrual cycle following oocyte retrieval or afterwards. Propensity score matching was used to make the potential risk factors of the two groups comparable. Multivariable regression analysis was used to study the effect of the timing of FET on pregnancy outcomes in the entire cohort and propensity score-matched cohort, even in different controlled ovarian hyperstimulation protocol cohorts as subgroup analysis.

**Results:**

We obtained 1130 patients in immediate FET group and 998 patients in delayed FET group, and the average age of the two groups were 30.30 and 30.63. We showed that the immediate FET group were equivalent to delayed FET group in the entire cohort [clinical pregnancy rate (CPR), 61.0% versus 63.4%, adjusted odd ratio (OR), 0.939, 95% confidence interval (CI), 0.781–1.129; spontaneous abortion rate (SAR), 10.1% versus 12.6%, adjusted OR, 0.831, 95% Cl (0.628–1.098); live birth rate (LBR), 49.9% versus 49.2%, adjusted OR, 1.056, 95% Cl (0.883–1.263)]. The same results were obtained by *χ*^2^ test in the propensity score-matched cohort (CPR, 60.5% versus 63.5%; SAR, 11.6% versus 12.3%; LBR, 48% versus 49.3%) (*P* > 0.05). Subgroup analysis indicated that pregnancy outcomes of immediate FET were no difference to delayed FET in gonadotropin-releasing hormone agonist (GnRH-a) protocol (*P* > 0.05). The SAR of the immediate FET group were lower than that of the delayed FET group in GnRH antagonist protocol (adjusted OR, 0.645, 95% CI, 0.430–0.966) (*P* < 0.05), no differences were observed in CPR and LBR (*P* > 0.05).

**Conclusions:**

The pregnancy outcomes of immediate FET were no difference to delayed FET in high responder population undergoing freeze-all cycles.

## Background

Controlled ovarian hyperstimulation (COH) is the key step in in vitro fertilization and embryo transfer (IVF-ET). High ovarian response (HOR) refers to the abnormal sensitivity of the ovary to gonadotropin, which leads to simultaneous development of multiple follicles and increases the risk of ovarian hyperstimulation syndrome (OHSS) [1]. Supraphysiological steroid hormones during COH affect endometrial receptivity by changing the endometrial immune environment and gene expression [2–4], resulting in poor pregnancy outcomes [5]. Adopting a freeze-all strategy in HOR patients can greatly reduce the risk of OHSS and avoid the influence of COH on endometrial receptivity [6]. An increasing number of studies have confirmed that frozen embryo transfer (FET) has better pregnancy and perinatal outcomes than fresh embryo transfer [7–9].

However, the best time to perform FET following COH in HOR patients is controversial in clinical work. Postponement of FET may increase the anxiety of patients [10]; in the immediate FET cycle, poor endometrial receptivity or physical condition may not be fully recovered to the pre-stimulation state, which may affect pregnancy outcomes [11]. It is unclear whether the detrimental effects on endometrial receptivity caused by COH would be sustained over a long period of time, up to the subsequent menstrual cycle, especially in patients with HOR who are most affected by COH. Moreover, the use of different gonadotropin-releasing hormone (GnRH) analogues in the process of COH act on the pituitary in different ways [12], and it is controversial whether the timing of FET affects pregnancy outcomes in different COH protocols [11, 13].

Thus, this study aimed to investigate whether the FET timing affects pregnancy outcomes in HOR patients undergoing freeze-all cycles, whether different COH protocols affect pregnancy outcomes in the subsequent FET cycle, and to provide reference results for the HOR population to choose the optimal time to start FET.

## Materials and methods

### Study population and design

We conducted a retrospective cohort study including all patients from January 2015 to March 2019 at our reproductive medicine center and the study was conducted in accordance with ethical standards (2020PS006F); informed consent was obtained from all subjects. The inclusion criteria were as follows: (1) patients on their first IVF or intracytoplasmic sperm injection (ICSI) cycle who were diagnosed with HOR and adopted a freeze-all strategy. The diagnostic criteria for HOR was > 5000 pg/ml of estradiol on human chorionic gonadotropin (HCG) day or more than 15 oocytes retrieved [14, 15]; (2) three different selected GnRH analogues stimulation protocols, including short-acting GnRH agonist (GnRH-a) long protocol, long-acting GnRH-a long protocol, and GnRH antagonist (GnRH-ant) protocol; (3) women 20–45 years old; and (4) hormone replacement therapy (HRT) for endometrial preparation in the FET cycle. The exclusion criteria were as follows: (1) presence of uterine abnormalities; (2) patients with endometriosis and adenomyosis; (3) presence of autoimmune, endocrine, and metabolic diseases; (4) previous diagnosis of uterine adhesion; (5) patients with chromosomal abnormalities; (6) patients who underwent blastocyst biopsy for pre-implantation genetic testing; (7) patients using frozen donor semen; (8) patients using long-acting GnRH-a as pretreatment for FET after the freeze-all cycle; (9) patients with a natural cycle for endometrial preparation for FET; (10) patients with no embryo formation for FET; and (11) patients with ectopic pregnancies.

Patients were divided into the immediate FET group and the delayed FET group, which were defined as FET that took place within the first menstrual cycle following oocyte retrieval or afterwards.

### Ovarian stimulation protocol, endometrial preparation protocol, and luteal support

According to age, anti-Müllerian hormone, body mass index (BMI), number of antral follicles in bilateral ovaries, and prior response to stimulation, we can predict the HOR population and determine the initial dose of gonadotropin to prevent the occurrence of OHSS [16, 17]. All patients were treated with the following three COH protocols: the short-acting GnRH-a long protocol involved daily subcutaneous injection of 0.05 mg of short-acting GnRH-a triptorelin (Diphereline, 0.1 mg, IPSEN, France) at the middle luteal phase of the menstrual cycle as pituitary down-regulation for 14 days, and gonadotropin introduction at the subsequent menstruation; the long-acting GnRH-a long protocol involved a single-dose intramuscular injection of a quarter to a full dose (0.75–3.75 mg) of long-acting GnRH-a (Diphereline, 3.75 mg, IPSEN, France) on the second day of menstruation, with gonadotropin given 20–30 days later when the follicle diameter reached 3–5 mm; the flexible GnRH-ant protocol involved starting gonadotropin on the second day of menstruation, and GnRH antagonist (Cetrotide, Merck Serono, France) was added when the lead follicle reached 13–14 mm in diameter or when the estradiol was > 300 pg/ml. Follicle development was detected by transvaginal ultrasonography, and the dosage of gonadotropin was adjusted according to the different ovarian responses.

When the follicles reached a mean diameter of > 17 mm, final oocyte maturation was triggered. HCG or triptorelin was used alone or in combination in the GnRH-ant protocol. Only HCG was used for the trigger in the GnRH-a long protocol. Oocyte retrieval was performed 36 h after triggering by transvaginal ultrasound-guided aspiration. Cleavage stage embryo quality was evaluated at day three based on the Istanbul consensus to confirm extended embryo culture, and the day three embryos with a cell number of ≥7 and fragmentation of < 20% were graded as good quality [18]. Blastocyst morphology was evaluated in the morning of days five and six according to the Gardner criteria [19], and only blastocysts scoring 4BB or higher were graded as good quality. We adopted a freeze-all strategy for HOR patients to avoid the occurrence of OHSS. The patients were informed that they could chose the menstrual cycle following oocyte retrieval for FET all by themselves.

Hormone replacement therapy (HRT) was used for endometrial preparation in the FET cycle, with 4–8 mg of estradiol valerate (Progynova, Bayer, Germany) taken orally for at least ten days from the 3rd to 5th day of menstruation to promote the growth of endometrium. Ultrasonic examination should be completed before medication, when the thickness of the endometrium is < 6 mm and the drug can be used, otherwise the FET in this cycle will be cancelled. Vitrification and warming procedures were performed using Embryo Vitrification/Thawing Media (KITAZATO) according to the manufacturer’s instructions. Cleavage-stage embryos were warmed on the day before transfer, cultured for approximately 24 h, and then transferred. The blastocysts were warmed on the day of transfer, kept in culture for approximately one hour, and then transferred.

The luteal phase was supported by 90 mg per day of vaginal progesterone gel (Crinone, Fleet Laboratories Ltd., UK) administered vaginally, and estradiol was maintained at the original dose. Luteal support was continued until 11 weeks of gestational age.

### Statistical analysis

The primary outcome of our study was live birth rate (LBR). Secondary endpoints were clinical pregnancy rate (CPR) and spontaneous abortion rate (SAR). Clinical pregnancy was defined as the detection of a gestational sac through ultrasound imaging at seven weeks of gestational age [20]. In China, spontaneous abortion was defined as the loss of pregnancy spontaneously after clinical pregnancy and before 28 weeks of gestational age, and live birth was defined as the survival delivery after 28 weeks of gestational age.

As an observational study, multiple maternal and IVF characteristics were considered as potential risk factors that could moderate pregnancy outcomes, and the potential risk factors between the immediate and delayed FET groups were unbalanced distribution (Table 1). Thus, we used propensity score matching (PSM) to make the potential risk factors between the immediate and delayed FET groups balanced and comparable. We used 1:1 nearest-neighbor matching without replacement to compare the variables and tried the match tolerance value from 1 to 0 until *P* values of the variable between the two groups were 1.000. *χ*^2^ test was performed for comparison of the categorical variables of the immediate and delayed FET groups (Table 1).

**Table 1:** Baseline characteristics of immediate and delayed FET groups in the entire and propensity score-matched cohorts

Potential risk factors	Entire cohort (***n*** = 2128)		***P***-value	Propensity score-matched cohort* (***n*** = 1366)		***P****-*value
	Immediate FET^**a**^	Delayed FET^**b**^		Immediate FET^**a**^	Delayed FET^**b**^	
	(***n*** = 1130)	(***n*** = 998)		(***n*** = 683)	(***n*** = 683)	
Maternal age (years)			0.455			1.000
≤ 34	976 (86.4)	851 (85.3)		622 (91.1)	622 (91.1)	
35–37	114 (10.1)	101 (10.1)		48 (7.0)	48 (7.0)	
≥ 38	40 (3.5)	46 (4.6)		13 (1.9)	13 (1.9)	
BMI (kg/m^2^)			0.431			1.000
< 18.5	70 (6.2)	58 (5.8)		465 (68.1)	465 (68.1)	
18.5–24.9	750 (66.4)	641 (64.2)		22 (3.2)	22 (3.2)	
≥ 25	310 (27.4)	299 (30.0)		196 (28.7)	196 (28.7)	
Insemination method			0.056			1.000
IVF	814 (72.0)	681 (68.2)		490 (71.7)	490 (71.7)	
ICSI	316 (28.0)	317 (31.8)		193 (28.3)	193 (28.3)	
COH protocol			0.000			1.000
GnRH-ant protocol	485 (42.9)	433 (43.4)	0.273^**#**^	284 (41.6)	284 (41.6)	0.651^**#**^
HCG trigger	330 (68.0)	306 (70.7)		199 (70.1)	199 (70.1)	
GnRH-a trigger	137 (28.2)	105 (24.2)		79 (27.8)	79 (27.8)	
Double trigger	18 (3.7)	22 (5.1)		6 (2.1)	6 (2.1)	
Short acting GnRH-a protocol	566 (50.1)	434 (43.5)		335 (49.0)	335 (49.0)	
Long acting GnRH-a protocol	79 (7.0)	131 (13.1)		64 (9.4)	64 (9.4)	
Number of embryo transfer			0.000			1.000
1	265 (23.5)	416 (41.7)		208 (30.5)	208 (30.5)	
2	865 (76.5)	582 (58.3)		475 (69.5)	475 (69.5)	
Embryo stage			0.000			1.000
Cleavage stage	711 (62.9)	499 (50.0)		418 (61.2)	418 (61.2)	
Blastocyst stage	419 (37.1)	499 (50.0)		265 (38.8)	265 (38.8)	
Top or good quality embryo transfer	947 (83.3)	838 (84.0)	0.919	584 (85.5)	573 (83.9)	0.408
Cause of infertility						
Tubal factor	720 (63.7)	630 (63.1)	0.778	456 (66.8)	456 (66.8)	1.000
Ovulatory disorder	304 (26.9)	257 (25.8)	0.548	167 (24.5)	167 (24.5)	1.000
Male factor	452 (40.0)	420 (42.1)	0.329	273 (40.0)	273 (40.0)	1.000
Unexplained factor	48 (4.2)	46 (4.6)	0.686	16 (2.3)	16 (2.3)	1.000
Multiple pregnancies	216 (19.1)	137 (13.7)	0.001	106 (15.5)	112 (16.4)	0.658

**Abbreviations:** BMI, body mass index; COH, controlled ovarian hyperstimulation; FET, frozen embryo transfer; GnRH-a, gonadotrophin-releasing hormone agonist; GnRH-ant, gonadotrophin-releasing hormone antagonist; HRT, hormone replacement therapy; IVF, in vitro fertilization; ICSI, intracytoplasmic sperm injection. Data are presented as number (%)

^a^Immediate FET and ^b^Delayed FET means FET took place either within the first menstrual cycle following oocyte retrieval or afterwards

*The predictors of propensity score matching were maternal age, BMI, insemination method, COH protocol, number of embryo transferred, embryo stage, top or good embryo transfer, and cause of infertility. The match tolerance was set to 0.000001. Categorical variables were compared with *χ*^*2*^ test. ^**#**^*P* value of differences of different trigger drugs in GnRH-ant protocol

As the effect of FET timing on different COH protocols were controversial, subgroup analysis was performed. Multivariable logistic regression models were calculated on each COH cohort, with the timing of FET as the main exposure of interest. Potential risk factors entered into the multivariable regression model were those that showed clinical relevance or showed a univariate relationship with pregnancy outcomes. The included variables were carefully selected based on the number of events available to ensure the stability of the regression equation. Adjusted odds ratio (OR) and their 95% confidence interval (CI) were calculated to analyze the independent effect of immediate and delayed FET on the pregnancy outcomes.

Statistical significance was set at *P* < 0.05. All statistical analyses were performed using IBM SPSS 26.0 (IBM Corp., Armonk, NY, USA).

## Results

### Potential risk factors between immediate and delayed FET groups in the entire and propensity score-matched cohort

**Fig. 1:**
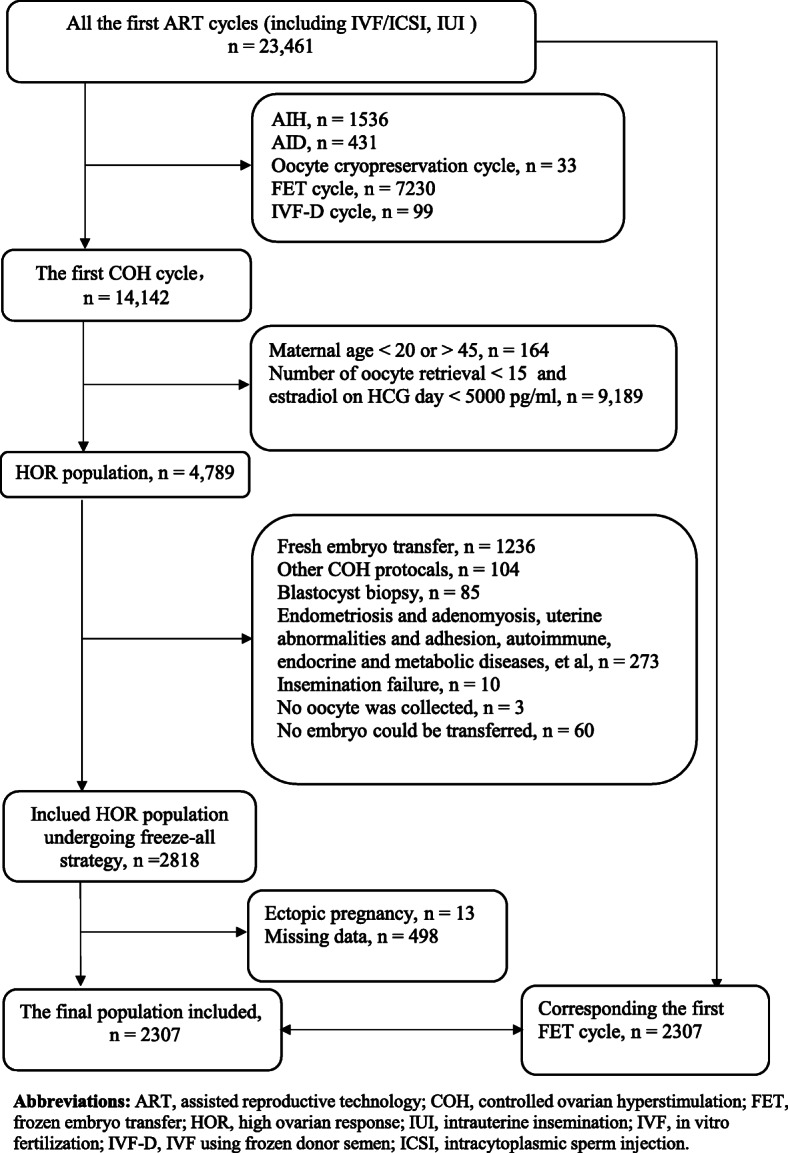
Flow chat showing the selection of the study polulation

A total of 2128 HOR patients adopting a freeze-all strategy underwent their first IVF/ICSI cycle (Fig. 1). The immediate and delayed FET groups consisted of 1130 and 998 patients, respectively. Patients’ and IVF characteristics in the immediate and delayed FET groups, which were the potential risk factors, are presented in Table 1. Before matching, the distribution of these risk factors were not absolutely balanced. The distribution of the COH protocol, number of embryos transferred, embryo stage, and multiple pregnancies were significantly different between the two groups (*P* < 0.05). No significant differences were found in maternal age, body mass index (BMI), insemination method, top or good quality embryo transfer, and infertility causes (*P* > 0.05). We obtained 1366 patients by PSM, and all potential risk factors and pregnancy outcomes of the multiple pregnancies were balanced and comparable (Table 1).

### Multivariable logistic regression analysis on FET timing and potential risk factors for pregnancy outcomes in the entire before-matching cohort

Multivariable logistic regression analysis on the entire before-matching cohort demonstrated no statistical differences on pregnancy outcomes between the immediate and delayed FET groups [CPR, adjusted odd ratio (OR), 0.939, 95% confidence interval (CI), 0.781–1.129; SAR, adjusted OR, 0.831, 95% Cl, 0.628–1.098; LBR, adjusted OR, 1.056, 95% Cl, 0.883–1.263] (*P* > 0.05), which were adjusted for maternal age, BMI, COH protocol, insemination method, number of embryos transferred, embryo stage, trigger type, top or good quality embryo transfer, and cause of infertility (Table 2).

**Table 2:** Multivariable logistic regression analysis on potential risk factors for pregnancy outcomes in the entire cohort

Potential risk factor variables	Adjusted OR* (95% CI)	***P****-*value
Clinical pregnancy		
FET timing (Immediate FET^a^ versus delayed FET^b^)	0.939 (0.781–1.129)	NS
Age (35–37 versus ≤34)	0.929 (0.690–1.251)	NS
Age (≥38 versus ≤34)	0.422 (0.268–0.663)	*P* < 0.001
BMI (< 18.5 versus 18.5–24.9)	1.073 (0.729–1.577)	NS
BMI (≥25 versus 18.5–24.9)	1.120 (0.910–1.378)	NS
COH protocol (short-acting GnRH-a versus GnRH-ant)	1.256 (1.010–1.562)	*P* < 0.05
COH protocol (long-acting GnRH-a versus GnRH-ant)	1.363 (0.973–1.909)	NS
Trigger type (GnRH-a trigger versus HCG trigger)	1.267 (0.924–1.738)	NS
Trigger type (Double trigger versus HCG trigger)	2.737 (1.227–6.106)	*P* < 0.05
Insemination method (ICSI versus IVF)	0.875 (0.687–1.115)	NS
Number of embryo transferred (2 versus 1)	1.573 (1.147–2.156)	*P* < 0.01
Embryo stage (blastocyst versus cleavage)	2.682 (1.999–3.599)	*P* < 0.001
Top or good quality embryo transfer (yes versus no)	1.203 (0.940–1.539)	NS
Tubal factor (yes versus no)	1.110 (0.877–1.407)	NS
Ovulatory disorder (yes versus no)	1.111 (0.877–1.407)	NS
Male factor (yes versus no)	1.172 (0.925–1.486)	NS
Unexplained factor (yes versus no)	1.452 (0.870–2.423)	NS
Spontaneous abortion		
FET timing (Immediate FET^a^ versus delayed FET^b^)	0.831 (0.628–1.098)	NS
Age (35–37 versus ≤34)	1.088 (0.693–1.707)	NS
Age (≥38 versus ≤34)	1.214 (0.638–2.310)	NS
BMI (< 18.5 versus 18.5–24.9)	0.967 (0.505–1.852)	NS
BMI (≥25 versus 18.5–24.9)	1.787 (1.336–2.391)	*P* < 0.001
COH protocol (short-acting GnRH-a versus GnRH-ant)	0.833 (0.594–1.167)	NS
COH protocol (long-acting GnRH-a versus GnRH-ant)	1.236 (0.758–2.014)	NS
Trigger type (GnRH-a trigger versus HCG trigger)	0.871 (0.550–1.379)	NS
Trigger type (Double trigger versus HCG trigger)	1.465 (0.642–3.343)	NS
Insemination method (ICSI versus IVF)	1.114 (0.768–1.617)	NS
Number of embryo transferred (2 versus 1)	0.965 (0.624–1.490)	NS
Embryo stage (blastocyst versus cleavage)	1.200 (0.769–1.810)	NS
Top or good quality embryo transfer (yes versus no)	1.114 (0.754–1.647)	NS
Tubal factor (yes versus no)	0.729 (0.514–1.033)	NS
Ovulatory disorder (yes versus no)	1.335 (0.949–1.878)	NS
Male factor (yes versus no)	0.709 (0.491–1.022)	NS
Unexplained factor (yes versus no)	0.744 (0.347–1.596)	NS
Live birth		
FET timing (Immediate FET^a^ versus delayed FET^b^)	1.056 (0.883–1.263)	NS
Age (35–37 versus ≤34)	0.936 (0.701–1.251)	NS
Age (≥38 versus ≤34)	0.365 (0.222–0.599)	*P* < 0.001
BMI (< 18.5 versus 18.5–24.9)	1.085 (0.748–1.574)	NS
BMI (≥25 versus 18.5–24.9)	0.841 (0.688–1.028)	NS
COH protocol (short-acting GnRH-a versus GnRH-ant)	1.267 (1.023–1.569)	*P* < 0.05
COH protocol (long-acting GnRH-a versus GnRH-ant)	1.241 (0.895–1.720)	NS
Trigger type (GnRH-a trigger versus HCG trigger)	1.316 (0.968–1.789)	NS
Trigger type (Double trigger versus HCG trigger)	1.956 (1.002–3.821)	NS
Insemination method (ICSI versus IVF)	0.892 (0.704–1.129)	NS
Number of embryo transferred (2 versus 1)	1.460 (1.092–1.953)	*P* < 0.05
Embryo stage (blastocyst versus cleavage)	2.289 (1.748–2.998)	*P* < 0.001
Top or good-quality embryo transfer (yes versus no)	1.224 (0.961–1.559)	NS
Tubal factor (yes versus no)	1.229 (1.032–1.635)	*P* < 0.05
Ovulatory disorder (yes versus no)	0.951 (0.757–1.195)	NS
Male factor (yes versus no)	1.326 (1.054–1.668)	*P* < 0.05
Unexplained factor (yes versus no)	1.549 (0.935–2.518)	NS

**Abbreviations:** BMI, body mass index; FET, frozen embryo transfer; GnRH-a, gonadotrophin-releasing hormone agonist; GnRH-ant, gonadotrophin-releasing hormone antagonist; IVF, in vitro fertilization; ICSI, intracytoplasmic sperm injection; OR, odds ratio; CI, confidence interval; NS, not significant

^a^Immediate FET and ^b^delayed FET means FET took place either within the first menstrual cycle following oocyte retrieval or afterwards

*Using the multivariable logistic regression and adjusting for maternal age, BMI, insemination method, COH protocol, trigger type, number of embryo transferred, embryo stage, top or good quality embryo transfer and cause of infertility

### Immediate versus delayed FET cycles on pregnancy outcomes in the entire cohort and propensity score-matched cohort

The CPR (entire cohort, 61.0% versus 63.4%; PSM cohort, 60.5% versus 63.5%), SAR (entire cohort, 12.6% versus 10.1%; PSM cohort, 11.6% versus 12.3%), and LBR (entire cohort, 49.9% versus 49.2%; PSM cohort, 48.0% versus 49.3%) had no significant differences between the immediate and delayed FET groups in the entire cohort and PSM cohort (*P* > 0.05) (Table 3).

**Table 3:** Pregnancy outcomes of immediate FET^**a**^ and delayed FET^**b**^ groups in the entire cohort and propensity score-matched cohort

Pregnancy outcomes	Entire cohort (*n* = 2128)		*P-*value	Propensity score-matched cohort (*n* = 1366)		*P-*value
	Immediate FET^a^	Delayed FET^b^		Immediate FET^a^	Delayed FET^b^	
	(*n* = 1130)	(*n* = 998)		(*n* = 683)	(*n* = 683)	
Clinical pregnancy	**61%**	63.4%	0.244	413 (60.5)	434 (63.5)	0.242
Spontaneous abortion	12.6%	10.1%	0.065	79 (11.6)	84 (12.3)	0.676
Live birth	49.9%	49.2%	0.743	328 (48.0)	337 (49.3)	0.626

**Abbreviations:** FET, frozen embryo transfer

^a^Immediate FET and ^b^Delayed FET means FET took place either within the first menstrual cycle following oocyte retrieval or afterwards. Data are presented as number (%)

Differences between the groups were evaluated by *χ*^2^ test.

### Subgroup analysis on immediate versus delayed FET on pregnancy outcomes in different COH protocol cohorts

To investigate the effect of FET timing on pregnancy outcomes in different COH protocols, multivariable logistic regression were performed on each COH protocol cohort, including 918 patients in the antagonist protocol, 1000 patients in the short-acting GnRH-a long protocol, and 210 patients in the long-acting GnRH-a long protocol. Multivariable logistic regression analysis demonstrated no statistical differences on pregnancy outcomes between the immediate and delayed FET groups in the short acting and long acting GnRH-a long protocols (*P* > 0.05). However, the SAR of the immediate FET group was lower than that of the delayed FET group in the GnRH-ant protocol (adjusted for maternal age, BMI, trigger type, insemination method, embryo stage, number of embryos transferred, top or good quality embryo transfer, and cause of infertility) (adjusted OR, 0.645, 95% CI, 0.430–0.966) (*P* < 0.05). No significant differences were found on CPRs and LBRs in the GnRH-ant protocol (*P* > 0.05) (Tables 4, 5, 6).

**Table 4:** Multivariable logistic regression analysis on potential risk factors for pregnancy outcomes in GnRH-ant protocol (*n* = 918)

Potential risk factor variables	Adjusted OR* (95% CI)	***P****-*value
Clinical pregnancy		
FET timing (Immediate FET^a^ versus delayed FET^b^)	0.956 (0.721–1.267)	NS
Age (35–37 versus ≤34)	0.798 (0.502–1.269)	NS
Age (≥38 versus ≤34)	0.386 (0.210–0.708)	*P* < 0.01
BMI (< 18.5 versus 18.5–24.9)	0.781 (0.424–1.440)	NS
BMI (≥25 versus 18.5–24.9)	0.981 (0.731–1.315)	NS
Trigger type (GnRH-a trigger versus HCG trigger)	1.229 (0.891–1.695)	NS
Trigger type (Double trigger versus HCG trigger)	2.560 (1.143–5.737)	*P* < 0.05
Insemination method (ICSI versus IVF)	1.006 (0.696–1.453)	NS
Number of embryo transferred (2 versus 1)	1.726 (1.040–2.865)	*P* < 0.05
Embryo stage (blastocyst versus cleavage)	3.062 (1.871–5.011)	*P* < 0.001
Top or good quality embryo transfer (yes versus no)	0.984 (0.647–1.497)	NS
Tubal factor (yes versus no)	1.043 (0.745–1.461)	NS
Ovulatory disorder (yes versus no)	1.242 (0.909–1.695)	NS
Male factor (yes versus no)	1.012 (0.720–1.423)	NS
Unexplained factor (yes versus no)	1.268 (0.622–2.585)	NS
Spontaneous abortion		
FET timing (Immediate FET^a^ versus delayed FET^b^)	0.645 (0.430–0.966)	*P* < 0.05
Age (35–37 versus ≤34)	1.202 (0.620–2.329)	NS
Age (≥38 versus ≤34)	1.511 (0.696–3.280)	NS
BMI (< 18.5 versus 18.5–24.9)	0.716 (0.245–2.095)	NS
BMI (≥25 versus 18.5–24.9)	1.400 (0.934–2.100)	NS
Trigger type (GnRH-a trigger versus HCG trigger)	0.907 (0.570–1.445)	NS
Trigger type (Double trigger versus HCG trigger)	1.408 (0.613–3.232)	NS
Insemination method (ICSI versus IVF)	1.274 (0.756–2.144)	NS
Number of embryo transferred (2 versus 1)	1.036 (0.553–1.943)	NS
Embryo stage (blastocyst versus cleavage)	1.537 (0.830–2.845)	NS
Top or good quality embryo transfer (yes versus no)	0.971 (0.538–1.753)	NS
Tubal factor (yes versus no)	0.847 (0.550–1.389)	NS
Ovulatory disorder (yes versus no)	1.583 (1.017–2.465)	*P* < 0.05
Male factor (yes versus no)	0.793 (0.483–1.304)	NS
Unexplained factor (yes versus no)	0.978 (0.353–2.710)	NS
Live birth		
FET timing (Immediate FET^a^ versus delayed FET^b^)	1.220 (0.925–1.608)	NS
Age (35–37 versus ≤34)	0.761 (0.479–1.210)	NS
Age (≥38 versus ≤34)	0.249 (0.121–0.514)	*P* < 0.001
BMI (< 18.5 versus 18.5–24.9)	0.883 (0.484–1.609)	NS
BMI (≥25 versus 18.5–24.9)	0.798 (0.599–1.063)	NS
Trigger type (GnRH-a trigger versus HCG trigger)	1.250 (0.916–1.707)	NS
Trigger type (Double trigger versus HCG trigger)	1.867 (0.951–3.664)	NS
Insemination method (ICSI versus IVF)	0.939(0.655–1.347)	NS
Number of embryo transferred (2 versus 1)	1.637 (1.027–2.610)	*P* < 0.05
Embryo stage (blastocyst versus cleavage)	2.409 (1.533–3.785)	*P* < 0.001
Top or good quality embryo transfer (yes versus no)	1.052 (0.701–1.580)	NS
Tubal factor (yes versus no)	1.144 (0.825–1.588)	NS
Ovulatory disorder (yes versus no)	0.994 (0.734–1.345)	NS
Male factor (yes versus no)	1.154 (0.827–1.611)	NS
Unexplained factor (yes versus no)	1.232 (0.619–2.450)	NS

**Abbreviations:** BMI, body mass index; FET, frozen embryo transfer; GnRH-a, gonadotrophin-releasing hormone agonist; GnRH-ant, gonadotrophin-releasing hormone antagonist; IVF, in vitro fertilization; ICSI, intracytoplasmic sperm injection; OR, odds ratio; CI, confidence interval; NS, not significant

^a^Immediate FET and ^b^delayed FET means FET took place either within the first menstrual cycle following oocyte retrieval or afterwards. *Using the multivariable logistic regression and adjusting for maternal age, BMI, insemination method, trigger type, number of embryo transferred, embryo stage, top or good quality embryo transfer, and cause of infertility

**Table 5:** Multivariable logistic regression analysis on risk factors for pregnancy outcomes in short-acting GnRH-a long protocol (*n* = 1000)

Potential risk factor variables	Adjusted OR* (95% CI)	***P****-*value
Clinical pregnancy		
FET timing (Immediate FET^a^ versus delayed FET^b^)	0.985 (0.751–1.294)	NS
Age (35–37 versus ≤34)	0.987 (0.636–1.530)	NS
Age (≥38 versus ≤34)	0.414 (0.194–0.879)	*P* < 0.05
BMI (< 18.5 versus 18.5–24.9)	1.391 (0.792–2.442)	NS
BMI (≥25 versus 18.5–24.9)	1.278 (0.918–1.778)	NS
Insemination method (ICSI versus IVF)	0.727 (0.508–1.040)	NS
Number of embryo transferred (2 versus 1)	1.401 (0.911–2.155)	NS
Embryo stage (blastocyst versus cleavage)	2.318 (1.595–3.370)	*P* < 0.001
Top or good quality embryo transfer (yes versus no)	1.148 (0.824–1.598)	NS
Tubal factor (yes versus no)	1.109 (0.767–1.603)	NS
Ovulatory disorder (yes versus no)	0.934 (0.634–1.375)	NS
Male factor (yes versus no)	1.229 (0.849–1.780)	NS
Unexplained factor (yes versus no)	2.101 (0.888–4.971)	NS
Spontaneous abortion		
FET timing (Immediate FET^a^ versus delayed FET^b^)	1.311 (0.826–2.080)	NS
Age (35–37 versus ≤34)	1.202 (0.604–2.392)	NS
Age (≥38 versus ≤34)	1.047 (0.297–3.699)	NS
BMI (< 18.5 versus 18.5–24.9)	1.246 (0.506–3.067)	NS
BMI (≥25 versus 18.5–24.9)	2.693 (1.681–4.314)	*P* < 0.001
Insemination method (ICSI versus IVF)	1.069 (0.567–2.015)	NS
Number of embryo transferred (2 versus 1)	0.856 (0.433–1.693)	NS
Embryo stage (blastocyst versus cleavage)	0.853 (0.470–1.547)	NS
Top or good quality embryo transfer (yes versus no)	1.380 (0.764–2.495)	NS
Tubal factor (yes versus no)	0.439 (0.229–0.841)	*P* < 0.05
Ovulatory disorder (yes versus no)	0.863 (0.459–1.621)	NS
Male factor (yes versus no)	0.349 (0.173–0.706)	*P* < 0.01
Unexplained factor (yes versus no)	0.225 (0.046–1.102)	NS
Live birth		
FET timing (Immediate FET^a^ versus delayed FET^b^)	0.929 (0.714–1.209)	NS
Age (35–37 versus ≤34)	0.968 (0.634–1.478)	NS
Age (≥38 versus ≤34)	0.430 (0.195–0.949)	*P* < 0.05
BMI (< 18.5 versus 18.5–24.9)	1.263 (0.743–2.150)	NS
BMI (≥25 versus 18.5–24.9)	0.856 (0.625–1.173)	NS
Insemination method (ICSI versus IVF)	0.797 (0.562–1.130)	NS
Number of embryo transferred (2 versus 1)	1.362 (0.913–2.033)	NS
Embryo stage (blastocyst versus cleavage)	2.283 (1.613–3.231)	*P* < 0.001
Top or good quality embryo transfer (yes versus no)	1.134 (0.820–1.569)	NS
Tubal factor (yes versus no)	1.458 (1.017–2.091)	*P* < 0.05
Ovulatory disorder (yes versus no)	0.920 (0.631–1.341)	NS
Male factor (yes versus no)	1.581 (1.105–2.262)	*P* < 0.05
Unexplained factor (yes versus no)	2.914 (1.306–6.500)	*P* < 0.01

**Abbreviations:** BMI, body mass index; FET, frozen embryo transfer; GnRH-a, gonadotrophin-releasing hormone agonist; IVF, in vitro fertilization; ICSI, intracytoplasmic sperm injection; OR, odds ratio; CI, confidence interval; NS, not significant

^a^Immediate FET and ^b^delayed FET means FET took place either within the first menstrual cycle following oocyte retrieval or afterwards

*Using the multivariable logistic regression and adjusting for maternal age, BMI, insemination method, number of embryo transferred, embryo stage, top or good quality embryo transfer, and cause of infertility

**Table 6:** Multivariable logistic regression analysis on risk factors for pregnancy outcomes in long-acting GnRH-a long protocol

Potential risk factor variables	Adjusted OR* (95% CI)	***P****-*value
Clinical pregnancy		
FET timing (Immediate FET^a^ versus delayed FET^b^)	0.607 (0.322–1.147)	NS
Age (35–37 versus ≤34)	1.218 (0.471–3.150)	NS
Age (≥38 versus ≤34)	0.810 (0.1052–6.257)	NS
BMI (< 18.5 versus 18.5–24.9)	0.980 (0.293–3.277)	NS
BMI (≥25 versus 18.5–24.9)	1.525 (0.715–3.256)	NS
Insemination method (ICSI versus IVF)	1.151 (0.463–2.862)	NS
Top or good quality embryo transfer (yes versus no)	8.805 (2.515–30.827)	*P* < 0.01
Embryo stage (blastocyst versus cleavage)	1.425 (1.163–5.056)	*P* < 0.05
Tubal factor (yes versus no)	2.454 (0.924–6.518)	NS
Ovulatory disorder (yes versus no)	0.840 (0.229–3.085)	NS
Male factor (yes versus no)	2.844 (1.147–7.051)	*P* < 0.05
Unexplained factor (yes versus no)	1.335 (0.204–8.721)	NS
Spontaneous abortion	**Adjusted OR**^**#**^ **(95% CI)**	
FET timing (Immediate FET^a^ versus delayed FET^b^)	0.380 (0.143–1.010)	NS
Age (35–37 versus ≤34)	0.409 (0.088–1.878)	NS
Age (≥38 versus ≤34)	–	NS
BMI (< 18.5 versus 18.5–24.9)	0.544 (0.066–4.491)	NS
BMI (≥25 versus 18.5–24.9)	1.914 (0.759–4.827)	NS
Top or good quality embryo transfer (yes versus no)	0.836 (0.207–3.375)	NS
Live birth	Adjusted OR* (95% CI)	***P****-***value**
FET timing (Immediate FET^a^ versus delayed FET^b^)	0.995 (0.543–1.823)	NS
Age (35–37 versus ≤34)	2.034 (0.800–5.168)	NS
Age (≥38 versus ≤34)	1.991 (0.248–15.991)	NS
BMI (< 18.5 versus 18.5–24.9)	1.290 (0.396–4.195)	NS
BMI (≥25 versus 18.5–24.9)	1.034(0.507–2.111)	NS
Insemination method (ICSI versus IVF)	1.422 (0.607–3.334)	NS
Top or good quality embryo transfer (yes versus no)	13.211 (2.621–66.596)	*P* < 0.01
Embryo stage (blastocyst versus cleavage)	2.339 (1.186–4.614)	*P* < 0.05
Tubal factor (yes versus no)	2.025 (0.842–4.868)	NS
Ovulatory disorder (yes versus no)	0.552 (0.148–2.057)	NS
Male factor (yes versus no)	1.470 (0.658–3.286)	NS
Unexplained factor (yes versus no)	0.397 (0.054–2.935)	NS

**Abbreviations:** BMI, body mass index; FET, frozen embryo transfer; GnRH-a, gonadotrophin-releasing hormone agonist; IVF, in vitro fertilization; ICSI, intracytoplasmic sperm injection; OR, odds ratio; CI, confidence interval; NS, not significant

^a^Immediate FET and ^b^delayed FET means FET took place either within the first menstrual cycle following oocyte retrieval or afterwards

*Using the multivariable logistic regression and adjusting for maternal age, BMI, insemination method, trigger type, embryo stage, top or good quality embryo transfer, and cause of infertility

^**#**^ Using the multivariable logistic regression and adjusting for maternal age, BMI, top or good quality embryo transfer

“-” means that the number of miscarriages in the group over 38 years old is 0.

## Discussion

In clinical work, the timing of when to start the FET cycle after COH is controversial, especially for patients with HOR who are most affected by COH. It is known that in the COH process, increased levels of supraphysiological steroid hormones and premature progesterone affects the gene expression and immune environment of the endometrium, which alters the embryo-endometrium asynchrony and negatively affects endometrial receptivity, reducing the CPR and LBR [5, 21]. Moreover, the secretory activity factors produced by the residual luteal cysts derived from COH last longer after oocyte retrieval in HOR patients. However, no study has investigated the specific duration of these adverse effects. To avoid this concern, some clinicians recommend the conservative scheme which is to start the FET cycle at the second or third withdrawal bleeding after oocyte retrieval. This prolongs the IVF treatment period and increase the anxiety of patients who had experienced infertility for many years, increasing their mental and economic loses and affecting their pregnancy outcomes [10, 22]. Lattes et al. reported that there were no differences on the first FET and subsequent FET cycles after oocyte retrieval [23]. A retrospective study by Huang et al. demonstrated that immediate FET was associated with a higher live birth than delayed FET [24], and meta-analysis indicated that immediate FET was not associated with negative pregnancy outcomes [25]. In our retrospective study, we only selected the HOR population who adopted HRT as the endometrial preparation protocol in the past five years and found no significant differences for CPR, SAR, and LBR between the immediate and delayed FET groups. We believe it is not accurate to assume that COH will still affect endometrial receptivity in the first withdrawal bleeding cycle after oocyte retrieval and that it is not necessary for the HOR population to wait for several menstrual cycles to begin FET after the freeze-all strategy.

In the process of COH, different GnRH analogues have different degrees and properties of inhibition effects on hypothalamic-pituitary-ovarian axis, and they also have different effects on the corpus luteum [26], which might have an impact on endometrial receptivity and pregnancy outcomes [27]. A retrospective study showed that immediate FET had similar CPR to delayed FET in patients with GnRH-ant protocol [13], which is in agreement with our subgroup results. However, our results are contrary to a population-based study on the short-acting GnRH-a long protocol, which found that delayed FET was better for pregnancy outcomes. They believed that the initial flare up effect of short acting GnRH-a during the down-regulation period caused an early rise of progesterone, which affected the outcomes in the immediate FET cycle [11]. However, a limitation of that study was the small sample size of 67 patients in the immediate FET group and 62 in the delayed FET group. In this study, 1000 patients with short acting GnRH-a long protocol were studied (434 in immediate FET group and 566 in delayed FET group), and we found that the timing of FET did not affect pregnancy outcomes in the short-acting GnRH-a long protocol. However, in the GnRH-ant protocol, we found that the SAR in the delayed FET group was significantly higher than that in the immediate FET group. Among all the COH protocols, the GnRH-ant protocol has the shortest treatment period, where oocyte retrieval takes place after a mean of 8–10 days of ovarian stimulation; the short-acting GnRH-a long protocol requires 14 days of down-regulation on that basis; while the long-acting GnRH-a long protocol needs a down-regulation of more than 20 days. Therefore, the immediate FET cycle in the GnRH-ant protocol can result in the shortest treatment period. Psychological factors can lead to infertility and spontaneous abortion, and its potential impact on neuroendocrine and immune changes could affect early pregnancy risk [28, 29]. We considered that longer treatment periods could result in increased patient anxiety, causing the increase in the SAR. It is worth mentioning that one study has shown that residual luteal cysts may increase the expression of relaxin in circulation [30], which is related to endometrial angiogenesis and prevents recurrent abortion [31]. Therefore, the effects of residual luteal cysts in immediate FET cycles that we had previously worried about may be beneficial to endometrial receptivity and pregnancy outcomes.

The limitation of this study lies in the retrospective nature as well as the possibility of unmeasured confounding factors such as smoking habits and alcohol consumption. Although we have obtained many cases with HOR and made the immediate and delayed FET groups comparable by PSM, we have lost 762 cases without successful matching in this process. In addition, we do not know whether these cases will affect the actual situation. Notably, the pregnancy outcomes we have studied were not the only endpoint, as other obstetric outcomes and neonatal outcomes should also be recorded. Moreover, the self-selection of patients into immediate or delayed FET remains a major limitation of the study. We believe that further prospective, randomized, and controlled studies are needed to confirm these results.

In summary, this study indicated that FET timing might not affect pregnancy outcomes in high responder patients undergoing freeze-all cycles. Immediate FET might be associated with lower odds of spontaneous abortion.

## Conclusion

The pregnancy outcomes of immediate FET were no different to delayed FET in high responder patients undergoing freeze-all cycles.

## Data Availability

The datasets generated and analysed during the current study were used under license for the current study, and so are not publicly available.

## Abbreviations

BMI: Body mass index
COH: Controlled ovarian hyperstimulation
CI: Confidence interval
CPR: Clinical pregnancy rate
FET: Frozen embryo transfer
GnRH-a: Gonadotropin-releasing hormone agonist
GnRH-ant: Gonadotropin-releasing hormone antagonist
HCG: human chorionic gonadotropin
HOR: High ovarian response
HRT: Hormone replacement therapy
ICSI: Intracytoplasmic sperm injection
IVF-ET: In vitro fertilization and embryo transfer
LBR: Live birth rate
OR: Odd ratio
OHSS: Ovarian hyperstimulation syndrome
PSM: Propensity score matching
SAR: Spontaneous abortion rate
